# Association of Baltic Sea and Mediterranean diets with frailty phenotype in older women, Kuopio OSTPRE-FPS study

**DOI:** 10.1007/s00394-020-02290-5

**Published:** 2020-05-27

**Authors:** Fatemeh Ramezan Alaghehband, Arja T. Erkkilä, Toni Rikkonen, Joonas Sirola, Heikki Kröger, Masoud Isanejad

**Affiliations:** 1 Institute of Public Health and Clinical Nutrition, University of Eastern Finland, 70211 Joensuu, Kuopio Finland; 2 Kuopio Musculoskeletal Research Unit, University of Eastern Finland, 70211 Joensuu, Kuopio Finland; 3 Department of Orthopaedics and Traumatology, Kuopio University Hospital, 70210 Joensuu, Kuopio Finland; 4 Institute of Life Course and Medical Sciences, University of Liverpool, Liverpool, L7 8TX England

**Keywords:** Baltic Sea diet, Mediterranean diet, Frailty, Older women

## Abstract

**Purpose:**

To evaluate the association between Baltic Sea diet (BSD) and Mediterranean diet (MED) with frailty.

**Methods:**

This was a secondary analysis on the osteoporosis risk factor and prevention–fracture prevention study on 440 women aged 65–72 years. Frailty was ascertained with the presence of 3–5 and prefrailty 1–2 of the following criteria: weight loss ≥ 5%, low life satisfaction score, walking speed ≤ 0.51 m/s, handgrip strength divided by body mass index ≤ 0.67 kg/m^2^ and physical activity ≤ 2 h/week. Women answered to questionnaires on lifestyle factors and 3-day food record. BSD score was ascertained using intake of nine and MED score of eight foods or nutrients components from food record. Multinomial logistic regression models adjusted for age, energy intake, smoking, living status, marital status and intervention group evaluated associations between MED and BSD with frailty phenotype status.

**Results:**

At 3-year follow-up, 206 women (46.8%) were prefrail and 36 (8.2%) were frail. After adjusting for confounders, a tendency was found between BSD per standard deviation (SD)-unit increase and lower likelihood of frailty (*β* = 0.62, 95% CI = 0.38–1.01, *P* = 0.057). Further, MED per SD-unit increase was associated with lower likelihood of prefrailty (*β* = 0.74, 95% CI = 0.6–0.9, *P* = 0.009). Consumption of vegetables was lower in frail (31.5 ± 36.0 g/day) and prefrail women (37.1 ± 42.0 g/day) than in non-frail women (48.6 ± 40.7 g/day) (*P* for trend = 0.041).

**Conclusions:**

Positive behavioral characteristics such as following MED and BSD may be associated with lower likelihood of prefrailty and frailty in older women. However, further longitudinal analyses are warranted.

**Electronic supplementary material:**

The online version of this article (10.1007/s00394-020-02290-5) contains supplementary material, which is available to authorized users.

## Introduction

Aging frailty poses decline in biological function that will gradually result in deterioration of physiological performance, reduction in the ability to respond to external stressors and an associated increase in vulnerability [1]. Frailty imposes considerable impact on health care cost and predicts various adverse health outcomes among older adults such as lower quality of life, elevated risk of falls, hospitalization, mobility disability, dependency, morbidity and mortality [2–4]. Although there are common pathways indicated for frailty, mechanisms underpinning frailty are still under investigation. Recently, the inadequate intakes of energy, protein, micro- and macronutrients, which are prevalent among older people, have been suggested as major drivers to onset and progression of frailty [5].

Researchers and care providers from different disciplines are avid to investigate mechanisms and strategies to tackle this unprecedented public health challenge [2, 3, 6]. It is now widely accepted that nutrition has a key role to prevent or reverse frailty. The important role of nutrition in the pathogenesis of frailty has been addressed in observational and interventional studies [7–9]. A healthy diet can provide foods and nutrients which in interaction can improve health outcomes in older people. Hence, studying the role of healthy dietary patterns is considered as a sensible approach. Adherence to a healthy diet is a significant behavioral characteristic which has been linked with reduced prevalence of chronic diseases and musculoskeletal health measures in older people [4, 10–14]. Recent systematic reviews and meta-analyses indicate that a healthy diet, which is rich in fruit, whole grain and vegetable is associated with lower likelihood of frailty [4, 15]. A common approach to capture healthy diet is via pre-defined dietary patterns such as Baltic Sea diet (BSD) and Mediterranean diet (MED) [16]. Few studies investigated the association between MED and frailty [4, 15, 17]; however, there is a dearth in research to compare the association of MED and BSD with frailty.

MED is characterized by being rich in plant-based food (such as fruit, vegetable, nut, cereal and legumes) and olive oil (as a main source of fat); low to moderate in fish, poultry, egg, dairy products and wine and low in red meat [17]. MED has been associated with decreased likelihood of various diseases like colorectal cancer [18], cardiovascular diseases [14] and diabetes [19] and might be associated with frailty [6, 17, 20, 21]. A systematic review argued that MED has been associated with lower risk of incident frailty as defined by Fried phenotype and frail scale [17]. Similarly, MED has been shown to be associated with decreased risk of frailty phenotype after 80 years of age [21]. More recently, an intervention study among 612 not frail or prefrail subjects from five European countries (UK, France, Netherlands, Italy and Poland) found that the higher adherence to MED was associated with decreased risk of frailty after 12 months of follow-up through modulation of microbiome [22].

Whereas, BSD has been characterized by high intake of Nordic fruits, roots, whole-grain products, rapeseed oil and fish and low consumption of processed meat products, red meat and alcohol [23]. BSD was originally developed to address the characteristics of a healthy diet in Nordic countries (Finland, Sweden, Norway and Denmark) [24]. Although a trend towards better health outcomes such as faster walking speed, less decline in relative skeletal muscle index and total lean mass [10], greater handgrip strength [25] and lower abdominal obesity [24] was observed by higher BSD diet scores, association between BSD and frailty has not been studied. The hypothesis of this study was that older women with higher score for BSD and MED have lower likelihood of frailty and prefrailty compared to their counterparts.

## Methods

### Study population

This study was a secondary data analysis on the data derived from the osteoporosis risk factor and prevention–fracture prevention study (OSTPRE-FPS) [26]. OSTPRE-FPS was a randomized population-based trial with a 3-year follow-up started in 2003 in Kuopio. The primary aim of the study was to determine whether vitamin D and calcium supplementation would be effective in preventing falls and fractures in postmenopausal women. The inclusion criteria of OSTPRE-FPS were age 65 years or older by the end of November 2002, willing to participate, living in Kuopio province and not participating in other bone densitometry studies. The participants of OSTPRE-FPS study were selected from population-based OSTPRE study [27]. The OSTPRE was a cohort study of *n* = 13,100 postmenopausal women born in 1932–1941 in the Kuopio region, Finland. The baseline postal invitation of OSTPRE-FPS study was sent to 5407 women. From this population, 3432 participants met all the inclusion criteria which were randomized into intervention (*n* = 1718) and control (*n* = 1714) groups by an independent statistician. Further, a subsample of 750 women (375 from each study group) was selected to undergo the clinical measurements and requested to fill the questionnaires including the 3-day food record [26]. From the subsample, there were 610 women from whom the physical function assessments and clinical measurements were collected. By the end of baseline study, 554 women returned a complete food record which allowed to calculate MED and BSD scores. Life satisfaction scale data (used as a surrogate of exhaustion in frailty definition) were missing for 114 of these women (missing data due to not answering the questionnaire or circling two answers). The final sample for this study was 440 (58% of 750) women that had all the components to compute MED and BSD scores and frailty phenotype. Comparison showed no significant differences for the baseline characteristics between the women included in this analytical sample and those excluded [7]. There was no statistical power analysis prior to this study, and it was calculated for the original intervention.

### Dietary intake

Women filled a food record for three consecutive days including 2 weekdays and 1 day at weekend at baseline. They had received the instruction of filling food records in advance and returned it during research visits. Subjects also reported the type of fat consumed on bread, in cooking, and baking separately. Uncertainties in diaries were clarified by phone call to the participants by a nutritionist [28]. Assessment of underreporting has previously been reported and none of the participants was excluded due to low energy intake [29]. Consumption of foods and the intake of energy and nutrients were calculated using Nutrica program (version 2.5, Finnish Social Insurance Institute, Turku, Finland). Frequency of consumption of alcohol portions was asked in a separate questionnaire; one portion of alcohol was considered as equal to 12 g.

### Baltic Sea diet score

The BSD score calculation has been explained and published in these data previously [10]. The BSD score was modified from the original definition by Kanerva et al. (2014) [16]. The final BSD score had nine components including the positive components: (1) total fruit and berries, (2) vegetables (root vegetables legumes and nuts, mushrooms and vegetable products—potato excluded), (3) fiber from total cereal products, (4) fish, (5) low fat milk (skim milk and milk with fat < 2%), (6) ratio of polyunsaturated fatty acids (PUFA) to saturated fatty acids (SFA), and negative components: (7) processed meat and sausage, (8) total fat intake as a percentage of total energy intake and (9) alcohol. The mentioned food and nutrients were categorized into quartiles. The first six food and nutrients were considered as positive components of BSD and received value zero for the lowest quartile and three for the highest quartile. Negative components of BSD score including (10) processed meat and sausage and (11) total fat intake (as a percentage of energy intake) received three for the lowest quartile and zero for the highest quartile. In addition, intake of alcohol ≥ 1 portion (12 g) per day received value one; otherwise, value zero was given. Mentioned scores were summed up and the final BSD score ranged from 0 to 25, higher score indicating higher conformance to BSD [10].

### Mediterranean diet score

MED score was ascertained based on the existing scores in literature particularly those studies that have applied MED score in Nordic cohorts [30–32] and it has been explained elsewhere in these data [10]. MED score had eight components including (1) root vegetables, legumes and nuts, mushrooms and vegetable product (potato excluded), (2) total fruit, (3) total cereal and potato, (4) fish, (5) PUFA + monounsaturated fatty acids (MUFA): SFA, (6) alcohol, (7) total meat including sausage and egg, and (8) total milk and dairy products. The first five food and nutrients were considered as positive components of MED. Intake of positive components equal or higher than median scored as one and intake of them lower than the median scored as zero. On the contrary, (7) total meat including sausage and egg, and (8) total milk and dairy product were negative components of MED. For the negative components, value zero was assigned to women whose consumption was equal or higher than the median, and one to the others. Since the moderate intake of alcohol is recommended in this dietary pattern, intake of 5–25 g alcohol per day received value one, and value zero was considered for intake of alcohol higher and lower than that range. Approximately, 63% of our subjects (*n* = 350) were non-consumers of legumes and the median intake of legumes was zero; we added legume to the vegetable group and not considered it as a separate group in defining MED. Finally, the values were summed up and ranged between 0 and 8, higher score reflecting the higher conformance to the MED [10].

### Frailty ascertainment

Frailty score was modified from the Fried frailty phenotype [33], which has been previously published in these data [7]. The criteria of this phenotype were (1) weight loss ≥ 5% of body weight over three years, (2) low life satisfaction score as surrogate for exhaustion (3) walking speed ≤ 0.51 m/s, (4) hand grip strength divided by body mass index (BMI) ≤ 0.67 kg/m^2^ to indicate weakness and (5) physical activity level ≤ 2 h/week. All the measurements were performed by trained nurses at Kuopio musculoskeletal research unit of clinical research center of University of Kuopio, Kuopio, Finland. The standard standing scale was used to measure weight (kg) in light indoor clothing twice (at baseline and after 3-year follow-up). Stadiometer was used to measure height (cm), BMI was calculated as kg/m^2^. To calculate the weight loss, we subtracted the measured crude weight (kg) at 3 years from the one measured at baseline and calculated the relative change in percentage. Women with ≥ 5% body weight loss received score of one, and otherwise zero. Unavailability of the unintentional or prior weight information for the women at baseline has limited our study to define modified frailty phenotype only at 3 years of follow-up. Life satisfaction score was used as a surrogate for exhaustion in Fried criteria [7]. In brief, life satisfaction was obtained using a four-item questionnaire [34]. The items in the questionnaire included current feelings of (1) interest in life, (2) happiness in life, (3) ease of living; and (4) loneliness. The scaled questions had four levels with two most opposite moods written at the separate ends of the scale and two additional options in the middle, which presented more intermediate levels of self-rated life satisfaction. Subjects showed their opinion by selecting one of the four options across the scale for each question. Total score ranged 4–20, higher score reflecting lower life satisfaction. Based on total life satisfaction score, subjects were categorized into three following groups: satisfied group (life satisfaction score: 4–6), intermediate group (life satisfaction score: 17–11) and dissatisfied group (life satisfaction score: 12–20). Dissatisfied group was considered as a surrogate for exhaustion in frailty ascertainment, and received score one, otherwise zero. To measure walking speed, women walked 10 m with their usual speed in a controlled situation. Time was recorded and walking speed was calculated as distance (m)/time (s). Walking speed was further divided for height and categorized into quartiles. Women in the lowest quartile ≤ 0.51 m/s of walking speed received score one, and otherwise zero. Handgrip strength (kg) was measured by pneumatic hand-held dynamometer (Sammons Preston, Bolingbrook, IL). Subjects were asked to sit on a chair and bend forearm of their non-dominant hand from elbow at 90-degree angle. The measurement was repeated 3 times with 30-s resting time in between and the strongest result was included. Further, grip strength was adjusted by dividing the grip strength value for BMI and women in the lowest quartile of handgrip strength received score one, and otherwise zero. Physical activity data were collected via a self-administered questionnaire where participants were asked to report the type, hours, and seasonality (winter and summer) of their physical activity. Types of physical activity included walking, cycling, skiing, swimming, aerobic exercise, sport balls, skating, floor ball, gymnastics and rowing. However, our data analyses showed that the main physical activities reported were skiing, walking, cycling, swimming and aerobic exercise, which explained over 90% of the weekly physical activity (data not shown), and accordingly they were used to compute the final physical activity variable in this study [7]. Weekly amount of physical activity during winter and summer was calculated and the average of them was used as an estimate of long-term physical activity. To indicate the low physical activity level, the computed physical activity was categorized into quartiles. Women in the lowest quartile had less than 2 h of physical activity per week which is less than World Health Organization recommendation for older people aged ≥ 65 years [35] and received score one and otherwise zero. To calculate the final frailty score, mentioned scores were summed up and each subject received a frailty score from 0 to 5. According to Fried criteria, we categorized women as frail with scores of 3–5, prefrail with scores 1–2 and those with score zero were considered as referent (non-frail).

### Selection of confounders

We selected the potential confounders a priori based on their reported association with frailty in the literature. Information about age, income (euro/month), marital status (unmarried, cohabiting, married, divorced and widow), living status (living alone, live with another person and live in retirement home), age at menopause, amount of physical activity (hour/week), number of chronic health disorders, medications, intake of alcohol (portion/week), smoking status (never, current and former smokers), and intake of self-supplementation for calcium and vitamin D were recorded from the postal questionnaire which was filled by women at baseline.

The association between mentioned confounders and BSD, MED and frailty was tested by multinomial logistic regression analysis. Since weight loss and low physical activity are criteria of frailty phenotype, and alcohol intake is component of BSD and MED, we did not consider weight, physical activity and alcohol intake as confounders to avoid over-adjustment. Age (years), living status, marital status and energy intake (kJ/day) were associated with frailty. We did not find any association between smoking status (current smokers, former smokers and never smokers) and frailty. However, we considered it as a confounder since it has been usually used as a covariate in literature [2, 3, 15]. Final statistical model to test associations was adjusted for age (years), living status, marital status, energy intake (kJ/day), smoking status and intervention group.

### Statistical analyses

Considering that around half of the study population were enrolled into calcium and vitamin D supplementation for about three years, the interaction terms between BSD and MED with vitamin D and calcium intervention were tested. There was no significant interaction by intervention or effect of intervention on the frailty. Thus, data were pooled for total population (intervention and control group). However, to exclude any possible effect of intervention on the association between diets and frailty status, the analyses were adjusted for intervention group. Main baseline characteristics of participants were compared across the frailty phenotype status (frail, prefrail and referent) using one-way ANOVA or alternatively nonparametric Kruskal–Wallis test for continuous variables and Chi-square test for categorical variables.

In the main analysis, BSD and MED were used as continuous variables, per standard deviation (SD)-unit increase and in tertiles. All the analyses were performed in adjusted and unadjusted models except for the univariate analysis which was conducted to calculate the means and standard deviations (SD) of BSD and MED according to frailty phenotype status in unadjusted model. The logistic regression analysis was applied to test the association between BSD and MED scores as continuous variables (prefrail and frail versus referent) and per SD-unit increase with frailty status (prefrail versus referent and frail versus referent). Multinomial logistic regression analysis tested the associations between tertiles of BSD and MED (the lowest BSD and MED tertile taken as a reference group) with frailty in three categories, frail, prefrail and referent. Odd ratio (β), standard error (SE) and 95% confidence interval (CI) were reported from regression analyses. Frailty was used as two categories (frail and prefrail vs. referent) in regression analysis to assess *P* for trend between tertile categories of BSD and MED and frailty status. Further, the associations between components of BSD and MED with frailty status were tested by analysis of covariance test (ANCOVA) followed by Tukey post hoc test. Mean, standard deviation (SD) and *P* value were calculated from these analyses. The associations between BMI with frailty status were tested by multinomial logistic regression analysis, where referent category was the recommended BMI range for elderly population in Finland (BMI = 24–29 kg/m^2^) [36]. All statistical analyses were conducted using IBM SPSS statistics software version 25. Statistical significance was set at *P* < 0.05.

## Result

The mean age of participants was 67.8 (SD 1.8) years, mean BMI was 28.8 (SD 4.7) kg/m^2^ and mean physical activity was 4.0 (SD 4.5) hour/week. At the 3-year follow-up, 206 (46.8%) women were identified as prefrail, while 36 (8.2%) were frail. Frail individuals were statistically significantly older and more likely to be alone at home as compared with prefrail and referent participants (Table 1). Alcohol consumption was significantly lower in frail women in comparison to prefrail and referent women; however, further analysis using alcohol based on MED recommendation (alcohol consumption categories 5–25 g alcohol vs. < 5 or > 25 g/day) with frailty status was not significant (data not shown).

**Table 1:** Baseline characteristics of participants according to frailty status

	Referent (*n* = 198)	Pre-frail (*n* = 206)	Frail(*n* = 36)	*P* value^a^
Age (year)	67.5 ± 1.8	68.1 ± 1.9	68.3 ± 1.9	0.010
Weight (kg)	69.2 ± 10.1	72.2 ± 12.5	77.8 ± 13.9	0.001
Height (cm)	159.4 ± 5.2	158.0 ± 5.7	156.7 ± 4.4	0.005
Body mass index (kg/m^2^)	27.2 ± 3.6	28.9 ± 4.9	31.7 ± 5.9	< 0.001
Income per month (euro)	904.1 ± 276.5	871.1 ± 315.5	781.8 ± 270.1	0.179
Duration of hormone therapy (year)	6.5 ± 6.7	6.0 ± 7.1	5.9 ± 7.8	0.757
Years since menopause (year)	17.6 ± 4.8	19.3 ± 5.1	19.3 ± 5.8	0.016
Alcohol (portions/ week)	1.0 ± 1.5	0.9 ± 1.5	0.4 ± 0.7	0.030
Current smoker *n* (%)	7 (3.7%)	11 (5.8%)	2 (6.7%)	0.867
Living status				0.029
Single living household *n* (%)	49 (24.9%)	66 (32.2%)	18 (51.47%)	
At home with another person *n* (%)	148 (75.1%)	136 (66.3%)	17 (48.6%)	
At retirement home *n* (%)	0 (0.0%)	2 (1.0%)	0 (0.0%)	
Marital status				0.084
Unmarried	9 (4.6%)	9 (4.4%)	3 (8.3%)	
Cohabiting	10 (5.1%)	12 (5.9%)	0 (0.0%)	
Married	131 (66.8%)	120 (58.5%)	16 (44.4%)	
Divorced	16 (8.2%)	16 (6.3%)	4 (11.1%)	
Widow	28 (14.3%)	49 (23.9%)	13 (36.1%)	

Categorical variables were presented as number of participants (percentages) and continuous variables as mean ± standard deviation (SD)

^a^*P* value from one-way ANOVA test and respective non-parametric test (Kruskal–Wallis) for continuous variables and Chi-square test for categorical variables

UNIANOVA analysis showed that MED score was significantly lower in frail and prefrail compared to referent group (*P* = 0.020), and pairwise comparison revealed that MED score was statistically significantly higher among referent women compared to prefrail women (*P* = 0.016), but not for BSD score (Table 2). The associations between components of BSD and MED with frailty status were not significant except that vegetable consumption (g/day) was lower in frail than prefrail and referent women (*P* = 0.041) (Table 2). Post hoc analysis showed that the prefrail group had significantly lower consumption of vegetables than the referent group (*P* = 0.024).

**Table 2:** Association between BSD and MED scores and components and frailty status

	Referent (*n* = 198)	Pre-frail (*n* = 206)	Frail (*n* = 36)	*P* value
BSD score^a^	13.3 ± 4.1	12.9 ± 4.0	11.3 ± 3.6	0.065
MED score^a^	4.3 ± 1.3	3.9 ± 1.3	4.0 ± 1.3	0.020
Components of BSD and MED diet				
Total fruit (g/day)	123.8 ± 114.0	118.6 ± 113.9	92.4 ± 77.4	0.463
Total fruits and berries (g/day)	189.2 ± 127.0	173.5 ± 115.2	124.5 ± 87.6	0.126
Vegetables (g/day)	48.6 ± 40.7	37.1 ± 42.0	31.5 ± 36.0	0.041
Fiber from cereal product (g/day)	14.8 ± 5.4	15.1 ± 5.3	14.2 ± 5.8	0.539
Total cereal and potato (g/day)	390.9 ± 115.5	377.7 ± 120.8	356.1 ± 94.9	0.902
Total milk and dairy (g/day)	896.5 ± 498.5	837.2 ± 457.6	875.2 ± 537.6	0.530
Low-fat milk (< 2%) (g/day)	803.2 ± 705.1	875.9 ± 651.5	715.5 ± 605.0	0.443
Total fat, energy (%)	31.1 ± 5.2	30.7 ± 6.0	31.4 ± 6.0	0.557
PUFA + MUFA: SFA	1.4 ± 0.5	1.2 ± 0.4	1.3 ± 0.4	0.101^b^
PUFA: SFA	0.5 ± 0.2	0.4 ± 0.2	0.4 ± 0.2	0.100^b^
Fish (g/day)	42.9 ± 44.8	41.5 ± 44.2	31.9 ± 33.6	0.728
Total meat and eggs (g/day)	93.4 ± 49.8	92.1 ± 48.7	97.0 ± 57.9	0.397
Processed meat (sausage) (g/day)	13.9 ± 22.1	16.6 ± 25.0	20.4 ± 22.2	0.129
Alcohol (g/day)	12.3 ± 19.0	11.0 ± 18.0	4.8 ± 8.7	0.139

Mean ± SD and *P* value were calculated using univariate ANOVA test

Covariates in models were age (years), energy intake (kJ/day), smoking status (never, current and former smokers), living status (living alone, live with another person and live in retirement home) marital status (unmarried, cohabiting, married, divorced, widow) and intervention group

^a^Unadjusted result is reported

^b^*P* value was determined using non-parametric Kruskal–Wallis test

The regression analysis showed that continuous BSD score was not associated with frailty (*P* = 0.209). However, there was significant inverse association between continuous MED score and frailty in both unadjusted (*β* = −0.143, SE = 0.019, *P* = 0.005) and adjusted (*β* = −0.138, SE = 0.020, *P* = 0.009) models. When expressing BSD and MED per SD increment, a tendency was observed between BSD per unit-SD increase and lower likelihood of frailty (*β* = 0.623, SE = 0.249, and *P* = 0.057) in an adjusted model (Table 3). Further, MED per unit-SD increase was associated with lower likelihood of prefrailty (*β* = 0.739, SE = 0.115, and *P* = 0.009) in an adjusted model (Table 3).

**Table 3:** Association between per SD-unit increase of Baltic Sea and Mediterranean dietary patterns and frailty status

	Referent	Prefrail (*n* = 206)			Frail (*n* = 36)		
		*β* (SE)	95% CI	*P* value	*β* (SE)	95% CI	*P* value
BSD per SD-unit increase	Ref	0.91 (0.11)	0.74–1.13	0.404	0.62 (0.25)	0.38–1.01	0.057
MED per SD-unit increase	Ref	0.74 (0.11)	0.59–0.93	0.009	0.84 (0.25)	0.51–1.38	0.492

*β* coefficient, standard of error (SE) and 95% confidence interval *CI* were measured using regression analysis

Model was adjusted for age (years), energy intake (KJ/day), smoking (never, current and former smokers), living status (living alone, live with another person and live in retirement home), marital status (unmarried, cohabiting, married, divorced, widow) and intervention group

**Fig. 1:**
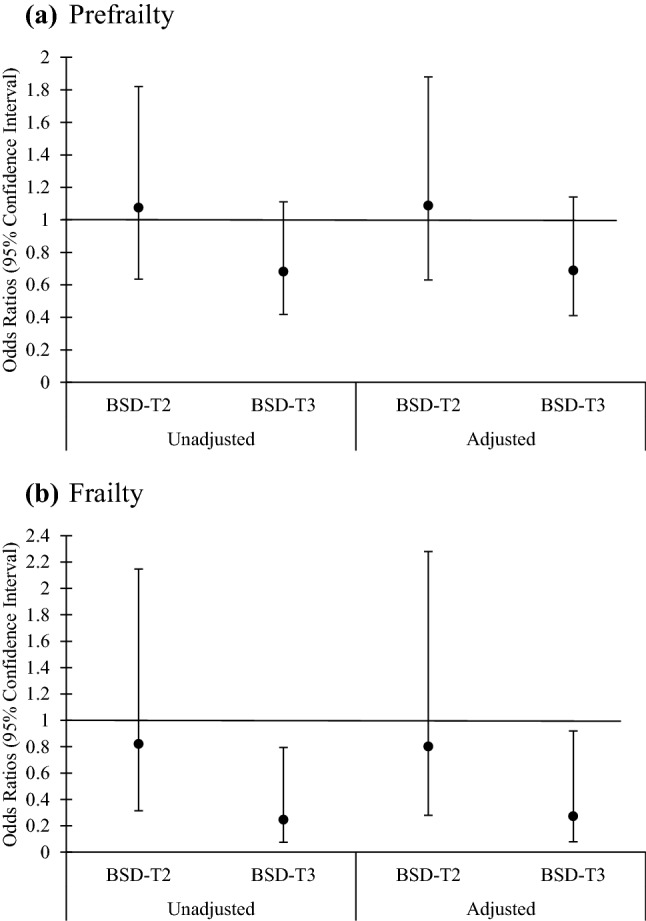
Likelihood of prefrailty and frailty for Baltic Sea diet tertiles (tertile 1 is referent = 1.0). Odds ratios and 95% confidence interval (Error bars represent the CI) were calculated using multinomial logistic regression analysis. Model was adjusted for age (years), energy intake (kJ/day), smoking status (never, current and former smokers), living status (living alone, live with another person and live in retirement home), marital status (unmarried, cohabiting, married, divorced, widow) and calcium and vitamin D supplementation intervention group. The reference category was referent (not frail). Unadjusted *P* for trend = 0.042. Adjusted *P* for trend = 0.066

**Fig. 2:**
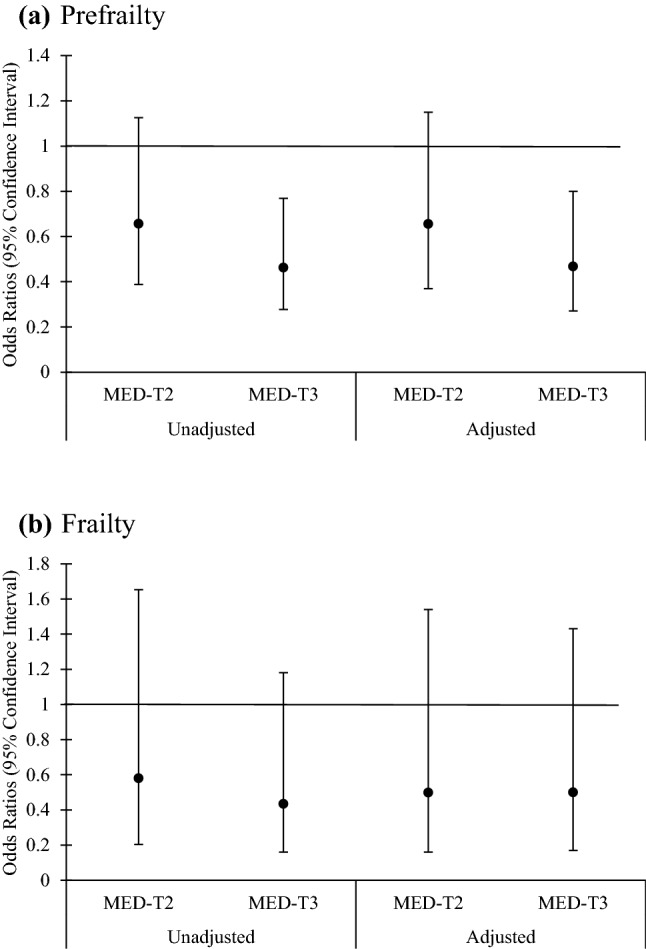
Likelihood of prefrailty and frailty for Mediterranean diet tertiles (tertile 1 is referent = 1.0). Odd ratios and 95% confidence interval (Error bars represent the CI) were calculated using multinomial logistic regression analysis. Model was adjusted for age (years), energy intake (kJ/d), smoking status (never, current and former smokers), living status (living alone, live with another person and live in retirement home), marital status (unmarried, cohabiting, married, divorced, widow) and calcium and vitamin D supplementation intervention group. The reference category was referent (not frail). Unadjusted *P* for trend = 0.005. Adjusted *P* for trend = 0.004

Figure 1 and Fig. 2 present the frailty and prefrailty likelihoods for MED and BSD tertile in multinomial logistic regression analysis. The frailty likelihood was lower in BSD tertile three compared to referent group in unadjusted (*β* = 0.246, CI = 0.076–0.794, *P* = 0.019) and adjusted models (*β* = 0.273, CI = 0.081–0.917, *P* = 0.036). However, the overall trend of association between tertile of BSD and frailty was only significant in unadjusted model (*β* = −0.104, CI = −0.030 to −0.001), with *P* for trend = 0.042 (Fig. 1). In Fig. 2 after adjusting for potential confounders likelihood for prefrailty (*β* = 0.468, CI = 0.273–0.801, *P* = 0.006) was lower in MED tertile three compared to referent group. In addition, the overall trend of association between MED tertile and frailty was significant in an adjusted model (*β* = -0.149, CI = −0.151 to −0.028, *P* for trend = 0.004). Among the confounders, we tested for the association between BMI and frailty in multinomial logistic regression analysis, where a strong statistically significant association was detected for BMI > 29 kg/m^2^ on frailty (*β* = 6.649, CI = 2.62–16.85, and *P* < 0.001) and prefrailty (*β* = 3.265, CI = 1.935–5.51, and *P* < 0.001) (Table 4).

**Table 4:** Association of BMI with frailty status

	Referent *n* = 198	Prefrail *n* = 206			Frail *n* = 36		
		*β* (SE)	95% CI	*P* value	*β* (SE)	95% CI	*P* value
BMI categories							
< 24 kg/m^2^, *n* = 118	Ref	1.44 (0.27)	0.85–2.44	0.174	0.60 (0.81)	0.12–2.95	0.528
24–29 kg/m^2^, *n* = 284	Ref	Ref	Ref		Ref	Ref	
> 29 kg/m^2^, *n* = 208	Ref	3.26 (0.27)	1.93–5.51	< 0.001	6.65 (0.47)	2.62–16.85	< 0.001

The referent was not frail

*β* coefficient, standard of error (SE) and 95% confidence interval *CI* were calculated using multinomial logistic regression analysis

Covariates in adjusted models were age (years), energy intake (kJ/d), smoking status (never, current and former smokers), living status (living alone, live with another person and live in retirement home), marital status (unmarried, cohabiting, married, divorced, widow) and vitamin D/ calcium intervention group

## Discussion

Findings of the current study showed that higher MED score was associated with lower likelihood of prefrailty and frailty in older women; whereas, we observed a tendency between higher BSD score and lower likelihood for frailty. These associations were independent of potential confounders. Regarding the BSD and MED food components, an inverse association was detected between vegetable intake and frailty likelihood after controlling for confounders.

Our results regarding inverse association of MED with frailty are consistent with the previous studies [4, 6, 17, 20, 21]. A recent systematic review has concluded that the higher conformance to MED is significantly associated with lower risk of incidence of frailty in community-dwelling older adults aged ≥ 60 years [17]. Ntanasi et al. (2018) also reported that the higher MED score is significantly associated with lower odds of frailty defined by three different methods (Fried criteria, frailty index and Tilburg frailty indicator) in 1740 participants older than 65 years [20]. Similarly, lower likelihood of frailty defined by frailty index was observed among participants who followed MED more closely than in those with less conformance in a prospective study with 8 years’ follow-up among 4421 North American subjects (mean age 61 years) [37]. In the French study, higher conformance to MED was associated with 62% lower risk of frailty in 560 older subjects, where frailty was ascertained according to Fried criteria [21]. Collected evidence from our study corroborate with existing research, suggest that MED is one healthy behavior which has been linked frequently with lower frailty likelihood, and it can be implemented for prevention efforts as well as in clinical practice.

Our study was the first to explore the association between BSD and frailty, where tendency was observed between BSD score per unit-SD increase and lower likelihood for frailty. The significant trend was observed between tertiles of BSD score and frailty and prefrailty. However, the significance was attenuated after controlling for confounders. Previous studies have indicated beneficial effect of BSD with various health outcomes [10], but there is paucity of data on association with frailty. Previously, in these data, higher conformance to BSD was shown to be associated with faster walking speed (m/s), less decline in relative skeletal muscle index (kg/m^2^) and total lean mass (kg) [10]. In addition, in Helsinki birth cohort study, higher conformance to BSD was significantly associated with greater hand grip strength in 1072 participants [25]. These results may somewhat explain the association between BSD and frailty via beneficial effect on physical function as an important component of frailty phenotype.

Further, our findings suggest that higher intake of vegetables (root vegetable, legume and nuts, mushrooms and vegetable products) was associated with lower likelihood of frailty, which was comparable to existing literature [8, 38, 39]. A systematic review study concluded that higher intake of fruit and vegetable might be associated with lower risk of frailty [38]. In another study with 22 years of follow-up, higher intake of fruit (*P* = 0.03) and vegetable (*P* = 0.009) was associated with lower risk of frailty, assessed by frail scale, in 30,267 women aged > 60 years [8]. An inverse dose–response relation was also found between intake of fruit (*P* = 0.04) and vegetable (*P* < 0.01) and both combined (*P* < 0.01) with frailty phenotype by Esquinas and coworkers (2016), who used random-effect meta-analysis to pool the result of three independent community-dwelling cohort studies with 1872, 581 and 473 subjects [39]. In general, MED and BSD are characterized with a high intake of fruit, vegetables, legumes, and low to moderate intake of alcohol and focus on consumption of high-quality fat [16, 17, 40]. In this study, intake of fruit and berries tended to be lower in frail and prefrail subjects; however, the association was not statistically significant. Different dietary assessment methods in the studies might explain the existing discrepancy between our result and previous evidence about the association between fruit consumption and frailty. The health benefits of fruits and vegetables are numerous and significantly associated with reduced risk of obesity [41], cardiovascular diseases [42], and bone mineral density [43]. Oxidative stress is considered as an important underlying factor of frailty among elderly [44], which may be modified by antioxidants in fruit and vegetables (vitamin C, vitamin E, carotenoid and selenium) [38, 39, 45], high content of polyphenols in fruits and vegetables can provide an anti-inflammatory properties to reduce the chronic inflammation and, therefore, reduce the cell apoptosis [39], which has been associated with a greater loss of muscle mass and strength, pronounced mobility loss, declined physical capacity, and depression in older individuals [46]. Recently, the metabolic role of vitamin E has been pronounced both as a strong free-radical scavenger and in pathways that are related to a higher energy metabolic phenotype and subsequently frailty [47]. Notwithstanding, the positive influence of MED and BSD on lower likelihood of frailty is not only attributed to high intake of fruit and vegetables, other nutrients profiles of BSD and MED, like unsaturated fatty acids, but also can contribute to anti-inflammatory properties [48]. Moreover, higher conformance to the BSD and MED has an important role in prevention of various chronic diseases such as impaired cognitive function [49], Alzheimer and dementia [13, 50], abdominal obesity [24, 51], depression [52] and diabetes [11, 19] which indirectly can slow the frailty progress [53–57]. Given the current knowledge, older people with higher conformance to MED and BSD are less likely to become chronically ill and consequently frail when compared to their counterparts [4, 17]. Although the mechanisms of gut microbial contribution to frailty are yet to be determined, adherence to healthy dietary patterns can improve gut microbiome and this microbiome response has the potential to attenuate the risk of frailty [22].

We also found a strong and significant association between higher BMI and increased likelihood of frailty (Table 4). Further analysis revealed that the subjects with BMI > 29 kg/m^2^ had significantly higher likelihood of frailty and prefrailty compared to those who had BMI between 24 and 29 kg/m^2^ (Table 4). This finding is in line with the previously published systematic review and meta-analysis among 16 studies which claimed that the obesity was associated with 43% higher risk of frailty [58].

The prevalence of frailty and prefrailty in this study (frail 8.1% and prefrail 46.8%) was comparable to previous studies, which report the prevalence rate of frailty between 7 and 30%, and prefrailty between 35 and 50% among community-dwelling older adults [2, 37, 59]. In a nutrition and health survey on 923 Taiwanese individuals aged 65 years or older, the prevalence of frailty and prefrailty was 7.8 and 50.8%, respectively [2]. Similarly, Veronese et al. found 8.2% frail among 4421 older adults (mean age of 61 years) in North America [37]. A Spanish cross-sectional study reported the proportion of 29.4% for frailty and 35.7% for prefrailty [59]. Use of various methods for identifying frailty and different characteristics of study participants such as age and gender can be the source of heterogeneity in the proportion of frailty and prefrailty. Although the prevalence rate of frailty in our study population is located on the lower end of mentioned range, we have considerable amount of prefrail women in our study population. Considering the fact that the frailty is a dynamic process with common transition from prefrailty to frailty [60, 61], frailty and prefrailty were merged as a single group to enhance the statistical power in some of the regression analyses.

There are limitations to this study which should be considered in interpretation of our findings. This was a cross-sectional study; therefore, reverse causality as frail or prefrail women altering their baseline dietary intake during the follow-up period cannot be excluded. Further, using life satisfaction scale as a surrogate of exhaustion might misclassify the participants in categories of frailty phenotype. Our study population included relatively healthy older women from a homogenous Finnish population; so, caution should be taken in generalization of the results to the other older populations. Other possible limitation was that computing dietary scores based on 3-day food record may not capture the consumption of infrequently consumed food [62]. Further studies could benefit to complement the self-reported dietary data with data on biomarkers of food and nutrient consumption or related risk factors. Half of our final analytical study population (*n* = 220) underwent the calcium and vitamin D supplementation intervention that possibly could indirectly affect awareness of participants in the intervention group over their dietary habits. However, the 3-day food record was only collected at the baseline; therefore, we cannot conclude neither if the changes in dietary patterns affect frailty nor the possible influence of supplementation intervention on dietary habits. In contrast to the original concept of BSD based on food frequency questionnaire measuring consumption frequencies of specified food groups, our fruit and meat categories based on food records included larger selection of individual food items. Further, our study did not consider legumes as a separate food group in defining MED score. The modified scoring of food groups for BSD and MED might have led to misclassification bias.

In conclusion, our results indicate that higher conformance to MED is associated with lower likelihood of prefrailty in older adults. In addition, our study showed that BSD may be presented as a healthy behavior to reduce likelihood of frailty. Amid BSD and MED components, only vegetables were inversely associated with frailty which suggests them to be a significant part of diet for inverse association with frailty. It is warranted that further studies need to focus to identify distinct frailty trajectories (clusters of individuals following a similar progression of frailty over time) in an elderly population and to estimate associations between frailty and frailty component trajectories and diet.

## Electronic supplementary material

Below is the link to the electronic supplementary material.

Supplementary file1 (DOCX 23 kb)

Supplementary file2 (DOCX 24 kb)
