# Supplementary material for: Association of Baltic Sea and Mediterranean diets with frailty phenotype in older women, Kuopio OSTPRE-FPS study
Source: Eur J Nutr. 2020 May 27;60(2):821–31. doi: 10.1007/s00394-020-02290-5 (PMC7900336; doi:10.1007/s00394-020-02290-5)
Supplement: Supplementary file 1 — Supplementary file1 (DOCX 23 kb) [file 394_2020_2290_MOESM1_ESM.docx]

| **Supplementary Table 1** Association of Baltic Sea diet score with frailty status | | | | | | | | | | | |
| --- | --- | --- | --- | --- | --- | --- | --- | --- | --- | --- | --- |
|  | **Referent**  **n=198** | **Prefrail**  **n=206** | |  | | **Frail**  **n=36** | | | **P _trend_**  **Unadjusted** | | **P _trend_ Adjusted ^a^** |
|  |  | β (SE) | 95% CI | |  | | β (SE) | 95% CI |  |  | |
| BSD Tertiles |  |  |  | |  | |  |  | 0.042 | 0.066 | |
| Tertile 1, n=183 | Ref | Ref |  | |  | | Ref |  |  |  | |
| Tertile 2, n=146 | Ref | 1.08 (0.28) | 0.63- 1.88 | |  | | 0.80 (0.53) | 0.28- 2.28 |  |  | |
| Tertile 3, n=178 | Ref | 0.69 (0.26) | 0.41- 1.14 | |  | | 0.27 (0.62) | 0.08- 0.92 |  |  | |
| β coefficient, standard of error (SE) and 95% confidence interval (CI) were calculated using multinomial logistic regression analysis. The reference category was Referent (not frail).  Tests for a linear trend across tertile of Baltic Sea diet score were conducted using the median value in each category as a continuous variable in the models in multinomial logistic regression analysis. Frailty was used as 2 categories (frail and prefrail vs. referent) in regression analysis to assess P for trend between tertile categories of diet and frailty status.  ^a^ Covariates in adjusted models were age (years), energy intake (kJ/d), smoking status, living status (living alone, live with another person and live in retirement home), marital status (unmarried, cohabiting, married, divorced, widow) and intervention group. | | | | | | | | | | | |
